# The Associations of Dental and Periodontal Lesions with the Microvascular Complications in Patients with Type 2 Diabetes Mellitus: A Case–Control Study

**DOI:** 10.3390/life14121585

**Published:** 2024-12-02

**Authors:** Adina Andreea Mirea, Adela Gabriela Ștefan, Moța Maria, Diana Clenciu, Adina Mitrea, Ion Cristian Efrem, Maria Magdalena Roșu, Diana Cristina Protasiewicz-Timofticiuc, Beatrice Elena Vladu, Theodora Claudia Gheonea, Felicia Mărășescu, Moța Eugen, Ionela Mihaela Vladu

**Affiliations:** 1 Department of Oro-Dental Prevention and Oral Health, Faculty of Dentistry, University of Medicine and Pharmacy of Craiova, 200349 Craiova, Romania; adinaturcu14@yahoo.com; 2 Calafat Municipal Hospital, 205200 Calafat, Romania; adela.firanescu@yahoo.com; 3 Doctoral School, University of Medicine and Pharmacy of Craiova, 200349 Craiova, Romania; mmota53@yahoo.com (M.M.); eugenmota@yahoo.com (M.E.); 4 Department of Diabetes, Nutrition and Metabolic Diseases, Faculty of Medicine, University of Medicine and Pharmacy of Craiova, 200349 Craiova, Romania; diana.clenciu@umfcv.ro (D.C.); theodora.gheonea@umfcv.ro (T.C.G.); ionela.vladu@umfcv.ro (I.M.V.); 5 Department of Medical Semiology, Faculty of Dentistry, University of Medicine and Pharmacy of Craiova, 200349 Craiova, Romania; 6 Department of Diabetes, Nutrition and Metabolic Diseases, Faculty of Midwives and Nursing, University of Medicine and Pharmacy of Craiova, 200349 Craiova, Romania; maria.rosu@umfcv.ro; 7 Department of Diabetes, Nutrition and Metabolic Diseases, County Clinical Emergency Hospital of Craiova, 200642 Craiova, Romania; diana_protasiewicz@yahoo.com; 8 Faculty of Medicine, University of Medicine and Pharmacy of Craiova, 200349 Craiova, Romania; beatricevladu75@gmail.com; 9 Department of Orthodontics, Faculty of Dentistry, University of Medicine and Pharmacy of Craiova, 200349 Craiova, Romania; ciuca_felicia@yahoo.com

**Keywords:** dental lesions, periodontal lesions, type 2 diabetes mellitus, microvascular complications

## Abstract

Background: Diabetes mellitus is closely related to periodontal disease and dental lesions, disorders which through dental infection and metabolic imbalance become negatively potentiated and cause a vicious circle that is almost impossible to break. The aim of this research was to study if the severity of dental and periodontal lesions is related to the presence of microvascular complications and glycemic control in patients with type 2 diabetes mellitus (T2DM). Methods: In total, 112 subjects with T2DM that underwent a dental evaluation were enrolled in this case–control study. The study group included 56 patients with complicated lesions, whereas the control group included 56 patients whose gender and age matched the study group and that presented superficial lesions. The statistical analysis was carried out using SPSS 26.0, with the result being considered statistically significant if the *p* values were <0.05. Results: Statistically significant differences were recorded between the two groups regarding the value of blood glucose, HbA1c and fibrinogen, as well as kidney function. Statistically significant differences were also recorded between the two groups when analyzing the presence of microvascular complications, as well as individually analyzed, in the case of diabetic peripheral sensory-motor neuropathy (*p* < 0.001), but also of diabetic retinopathy (*p* < 0.05). This study developed a score with a predictive value for the presence of complicated dental and periodontal lesions, including blood glucose, fibrinogen, diabetic retinopathy, and diabetic peripheral neuropathy (AUROC 0.847, *p* < 0.001). Conclusions: There is a high frequency of dental and periodontal complications in patients with T2DM. Patients with microvascular complications, elevated fasting blood glucose, and chronic inflammation, as evidenced by elevated fibrinogen, are more likely to develop complicated dental and periodontal lesions.

## 1. Introduction

Diabetes mellitus represents a worldwide major public health problem, having a significant impact on morbidity and mortality. With a prevalence that is constantly increasing and with hundreds of millions of people being affected by this disease, this metabolic impairment is one of the major causes of blindness, chronic kidney disease and non-traumatic amputations [1]. Type 2 diabetes mellitus (T2DM) determines a series of changes in the oral cavity, having a direct impact on the oro-dental status, but also on therapeutic interventions [2]. Thus, diabetes and periodontal disease and dental lesions are two closely related conditions, with major implications both in terms of the increased frequency of oral manifestations in patients with diabetes, as well as in terms of the ability of oral infections to cause metabolic imbalances. The two conditions are negatively potentiated, determining a vicious circle that is difficult to break [3,4].

The microvascular complications of diabetes mellitus, represented by neuropathy, nephropathy and retinopathy [1,5], are debilitating conditions, which many times imply a multidisciplinary approach [6]. On the other hand, severe oral complications, such as periodontitis, edentitis and temporomandibular joint dysfunction, also significantly reduce the quality of life in these patients [7]. The importance of this association is also emphasized by the fact that periodontal disease is considered the sixth most common complication of diabetes [8,9].

Numerous mechanisms involved in the development of periodontal disease in patients with diabetes have been cited. The first mechanism is that of polyols, which, through the enzyme aldose-reductase, transform glucose into sorbitol and through sorbitol dehydrogenase into fructose-1-phosphate. This first mechanism takes place in the cells of the large vessels at the neuronal level and in the cells of the Schwann’s sheath. The polyol pathway leads to the accumulation of the two osmotically active components, thus resulting in water retention, and therefore hydropic degeneration and later the appearance of diabetic neuropathy. A second mechanism cited in the development of changes in the oral cavity is represented by the formation of advanced glycation agents (AGEs), which is the main cause of chronic microvascular complications. This process consists of the binding of glucose to tissue proteins and blood, resulting in the formation of final products of advanced glycation, which cannot be eliminated, as they accumulate at the level of the vascular membrane. All these changes alter the structure and implicitly the function of the cell membrane. The accumulation of these final products of advanced glycation at the membrane level reduces the reactivity of neutrophils and causes a reduced oxygenation of the tissues, which implies a decrease in the immune response and delayed wound healing, as well as the occurrence of recurrent bacterial and fungal infections [2,10]. Other mechanisms possibly involved in increasing susceptibility to periodontal diseases include vascularization, collagen structure and metabolism, subgingival microflora and gingival crevicular fluid, but also genetic mechanisms. Moreover, risk factors are also involved, such as metabolic imbalance, diabetes duration, poor oral hygiene and smoking [11,12,13,14].

Regarding the higher frequency of dental lesions in patients with T2DM, although the association is generally recognized, the mechanisms that lead to dental lesions in these patients are still incompletely elucidated. Patients with diabetes are more likely to develop dental caries as they have hyposalivation, a result of both high carbohydrate ingestion and insulin deficiency, resulting in increased salivary glucose levels [15,16]. In this context, our study aims to investigate if the severity of dental and periodontal lesions in patients with T2DM is related to the glycemic control and the presence of microvascular complications.

## 2. Materials and Methods

### 2.1. Participants

**Figure 1:**
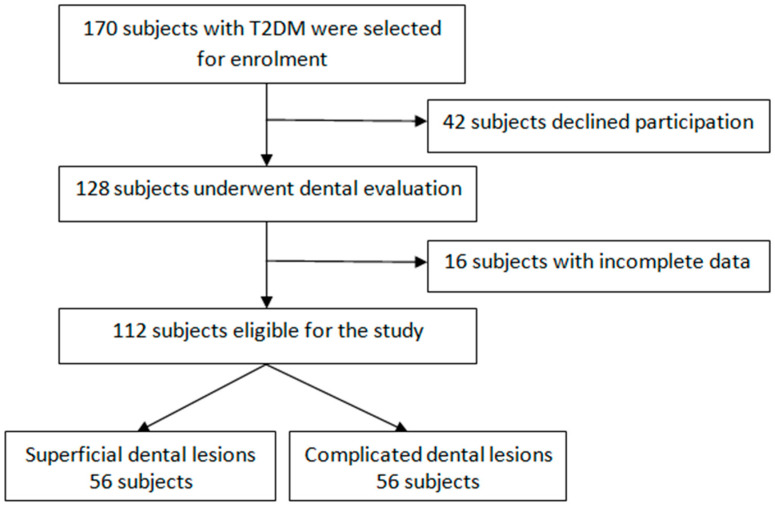
Study flow chart. T2DM: type 2 diabetes mellitus.

We carried out an epidemiological, non-interventional case–control study, which took place between February 2022 and January 2023, using the STROBE guidelines. The sample size was calculated using calculator.net [17], using a 95% confidence interval, which corresponds to a z-score of 1.96, with a margin of error of 5%. We assumed that a percentage of 30% of the patients would refuse to participate in the study, as well as a percentage of 15% of them would have incomplete data. Therefore, we estimated that 112 study participants should be enrolled in the study to meet the 95% confidence interval. The study flow chart is presented in Figure 1.

The inclusion criteria in the study included age, adult patients only (aged over 18 years old), patients diagnosed with T2DM and patients that underwent dental evaluation during their hospitalization at the County Clinical Emergency Hospital of Craiova. For the control group, further inclusion criteria, regarding gender and age, were also added, as they were age and gender matched to the study group. Regarding the exclusion criteria, subjects with ages under 18 years were excluded from the study, as well as patients without diabetes or diagnosed with type 1 diabetes or other types of diabetes. Furthermore, patients with other conditions that could influence the results of the investigations, such as smokers or patients with serious comorbidities (neoplasia of the oral cavity, substance abuse at the time of the study, or in their personal history, end-stage renal disease, liver insufficiency, etc.) were also excluded from this study.

A total of 112 patients were enrolled in the study: 56 with complicated dental and periodontal lesions (study group), and 56 with superficial dental and periodontal lesions (the control group) that had a similar age and gender. The subjects’ participation in the study was voluntary only after signing the informed consent forms. The study was conducted respecting the ethical principles stipulated in the Declaration of Helsinki and in accordance with Good Clinical Practice (GCP) guidelines, respecting the subjects’ right to confidentiality and integrity and giving every participant the option to withdraw from the study at any time.

We recorded the most significant data from the physical examination and the anamnesis. Demographic and anthropometric data (weight and height) were also registered. We calculated the body mass index (BMI) according to the formula BMI = weight (kilograms)/height squared (meters) and we interpreted the results according to the criteria of the World Health Organization (WHO) [18] for all the participants.

### 2.2. Laboratory Exams

Venous blood was collected from each subject in a vacutainer, using the standard procedure, and labeled with the identification number of the study participant. Fasting blood glucose, erythrocyte sedimentation rate (ESR), C-reactive protein and fibrinogen were also recorded. Glycated hemoglobin (HbA1c) was measured using the High-Performance Liquid Chromatography assay (HPLC) method, as well as serum creatinine [19]. Furthermore, we also determined albuminuria for the assessment of chronic kidney disease.

### 2.3. Evaluation of Microvascular Complications

The patients were screened for the presence of microvascular complications. The Toronto score was performed to assess diabetic peripheral neuropathy. Patients reporting symptoms of diabetic neuropathy in the lower limbs, as well as abnormal patellar and achillean reflexes or abnormal sensibility tests (TipTherm test for temperature perception, the 10 g monofilament for pressure perception, the Rydel–Seiffer tuning fork for vibration perception, the pricking test with neurotips and proprioception tests), were diagnosed with diabetic peripheral neuropathy according to the following criteria: (i) 1 point for each symptom present; (ii) 1 point for each method applied unsuccessfully, 1 point if the reflexes are diminished and 2 points if the reflexes are abolished; (iii) if the Toronto score was above 6 points, the diagnosis of peripheral diabetic neuropathy was established [20]. Diabetic retinopathy was evaluated by means of the fundus examination to detect possible microvascular anomalies [21]. The diagnosis of chronic kidney disease was established according to the Kidney Disease: Improving Global Outcomes (KDIGO) recommendations, using the estimated glomerular filtration rate (eGFR) calculated according to the Chronic Kidney Disease-Epidemiology Collaborative Group (CKD-EPI) formula [19,22].

### 2.4. Evaluation of Dental Lesions

**Figure 2:**
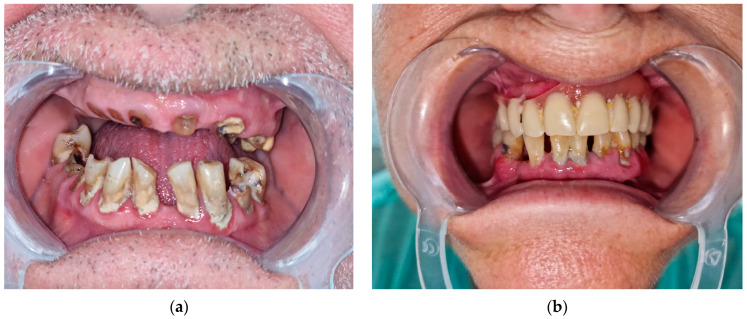
Complicated dental lesions in patients with type 2 diabetes mellitus: (**a**) Dental fractures; (**b**) periodontal disease.

In all the patients, a dental consultation was performed to establish the presence of dental and periodontal lesions, which were classified into superficial and complicated lesions according to national protocols derived from the Ellis classification [23,24]. Superficial lesions were defined as the presence of incipient caries, abfractions, cracks, erosions, abrasions and attrition. Patients with deep caries, fractures, abscesses, severe erosions, gingivitis and periodontal disease were diagnosed with complicated lesions (Figure 2).

### 2.5. Statistical Analysis

All the recorded data were registered in an Microsoft Excel 2019 database, which was later transferred to the Statistical Package for the Social Sciences (SPSS) version 26.0 (SPSS Inc., Chicago, IL, USA). The data were coded and analyzed according to the severity of the dental injuries. The Kolmogorov–Smirnov test was used to test the distribution of continuous variables. If the variables presented a normal distribution, they were reported as mean ± standard deviation (SD), while the ones with abnormal distribution were presented as median and interquartile range (IQR).

The tests used to determine the statistically significant differences between the two groups were the Student’s t-test to compare the means (for variables with normal distribution) and the Mann–Whitney U test to compare the medians (for variables with abnormal distribution). Categorical variables were analyzed using the chi-square test. The predictive value was assessed using the analysis of the area under the ROC curve (AUROC).

The results were considered statistically if the *p* value recorded was <0.05.

## 3. Results

The demographic, anthropometric and paraclinical characteristics of the subjects enrolled in the study are presented in Table 1.

The analysis of the subjects’ demographic characteristics showed no statistically significant difference between the two groups in terms of gender (*p* = 0.844) and age (*p* = 0.194). Moreover, when we analyzed the anthropometric parameters, we could not demonstrate a statistically significant difference between the study groups both when comparing BMI (*p* = 0.699), as well as nutritional status (*p* = 0.231). Regarding the laboratory markers, we assessed inflammatory biomarkers and observed that only in the case of the C-reactive protein value, the results did not reach statistical significance (*p* = 0.761), while both ESR (*p* = 0.001) and fibrinogen (*p* < 0.001) presented statistically significant higher values in patients with complicated dental lesions. The analysis of fasting plasma blood glucose and HbA1c, parameters associated with the T2DM control, showed highly significant differences (*p* < 0.001) between the two groups. Renal function was assessed by both eGFR and albuminuria, with both parameters showing statistically significant differences between the two study groups. Regarding the duration of T2DM since diagnosis, we observed that although the patients with complicated lesions presented a higher duration of diabetes, the *p* value did not reach statistical significance (*p* = 0.056). Moreover, highly statistically significant differences were also recorded in the case of subjects treated with insulin and metformin (*p* < 0.001), but also statistically significant differences regarding the treatment with the glucagon-like peptide 1 receptor agonist (GLP-1 RA) (*p* = 0.023), as it is presented in Table 1.

Table 2 presents the relationships between the microvascular complications of diabetes and the presence of dental and periodontal lesions in our study population. As can be observed, twice as many patients with diabetic retinopathy were diagnosed in the group with complicated lesions, with the difference reaching statistical significance (31.5% vs. 14.8%, *p* = 0.040). Moreover, almost all study participants who presented complicated lesions were diagnosed with diabetic peripheral sensory-motor neuropathy, with the difference between the two groups being highly statistically significant (*p* < 0.001) and with a frequency of 90.7% in subjects with complicated lesions and 61.1% in those with superficial lesions. Regarding chronic kidney disease, although twice as many were recorded in the group with complicated dental and periodontal lesions (22.2% vs. 9.3%), the difference did not reach statistical significance (*p* = 0.064).

We assessed the usefulness of the parameters that were associated with the presence of complicated lesions as predictors for the appearance of these lesions by using the AUROC analysis, as illustrated in Table 3. We considered the variables with AUROCs over 0.800 the most significant predictors of dental and periodontal complications in T2DM in our study group and calculated cut-off points for these variables, which were 240 mg/dL for fibrinogen (sensibility 89%; specificity 83%) and 149 mg/dL for fasting plasma glucose (sensibility 72%; specificity 56%).

**Figure 3:**
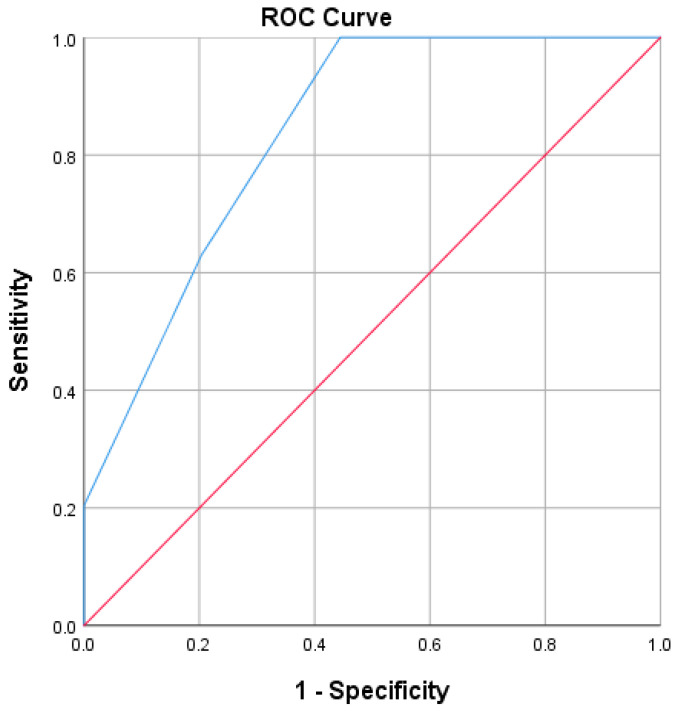
ROC curve for new score predicting complicated dental and periodontal lesions in patients with type 2 diabetes (AUROC: 0.847). Red line—reference line; Blue line—ROC curve for our score.

Taking into account the two predictive cut-off values resulted from the AUROC analysis and the presence of the microvascular complications associated with complicated dental and periodontal lesions in our study (diabetic retinopathy and diabetic peripheral neuropathy), we elaborated a score (Table 4) with four items (each item receiving 1 point) that presented an AUROC of 0.847 (standard error 0.037; CI 95%: 0.774–0.920), which provided a statistically significant (*p* < 0.001) predictive value for the presence of complicated lesions in our study (Figure 3).

## 4. Discussion

In recent years, the relationships between the oral lesions, metabolic syndrome, T2DM and the chronic complications of diabetes have been the subject of comprehensive studies, considering the impact of the condition on the oral status. Metabolic syndrome, a condition associated with T2DM, hyperlipidemia, metabolic dysfunction-associated liver steatotic disease, cardiovascular disease and cancer were also associated with periodontitis [25,26,27,28]. A possible explanation of this link could be the ailments from dental/peridental foci leading to a host immune inflammatory response [25,27]. On the other hand, early identification and adequate management of oral manifestations determine good glycemic control [2,29].

Regarding patients with oral complications, a significant association of peripheral sensory-motor diabetic neuropathy was found between the two disorders in numerous studies. In a study conducted in a group of 50 Indian patients with T2DM, it was demonstrated that the presence of diabetic neuropathy was linked with the presence of oral complications (*p* < 0.05). Moreover, the basal and postprandial glucose levels were higher among subjects with T2DM and oral complications (*p* < 0.05) [3,7]. In another study conducted in a group of Swedish subjects with T2DM and diabetic neuropathy, almost half of the studied patients had less than 10 natural teeth, compared to those without neuropathy [7,30].

Other authors analyzed a group of patients from Brazil with uncontrolled T2DM, with one third having moderate/severe periodontitis and over a quarter being edentulous. In addition, the presence of severe periodontitis was significantly associated with diabetic neuropathy. After adjustments were made for age, gender, dental health care, duration of T2DM, and education, it was demonstrated that plantar ulcers were an independent predictor for the severity of periodontitis and edentulousness [7,31]. Similarly, a team of researchers studied a group of Mexican subjects with T2DM, with more than 60% of them being edentulous. Furthermore, about 50% of the patients that presented severe periodontitis were also diagnosed with diabetic neuropathy [7,32]. Also, analyzing a narrow group of Finnish subjects, peripheral diabetic neuropathy and autonomic neuropathy were present at a statistically significant higher percentage in the patients with severe edentulousness, with the relationship between the two pathologies being demonstrated through logistic regression [7,33].

In a study carried out in 2012, Bajaj et al. [3] analyzed the higher frequency of microvascular complications in subjects with DM and oral complications, compared to those without oral complications, being also associated with higher values of fasting and postprandial glucose. The same relationship between fasting blood glucose and the severity of the dental lesions was observed in our study. Although postprandial blood glucose was not studied in our research, the importance of blood glucose control in reducing the frequency of severe dental lesions was proven by the fact that increased HbA1c was a predictor of complicated dental lesions. Numerous other authors have demonstrated an increased severity of periodontitis in people with poorly controlled DM and it is known that metabolic imbalance is a major determinant for the appearance of chronic complications of DM [3,34,35,36]. Moreover, our research confirms the results obtained by other studies that have proven a significant link between periodontal disease and micro-vascular complications of diabetes (retinopathy and neuropathy) [3].

A study published by Saremi et al. in 2005 presented the results of the analysis of a cohort followed for a median time of 11 years. They found that subjects with severe periodontal disease have a 3 times higher risk of cardio-renal mortality, compared to subjects with mild, moderate or absent periodontal disease [4,37]. Another important study published in 2007 by Shultis et al. highlighted a significant association between the severity of periodontal disease and edentulousness. Furthermore, this study showed an association between dental lesions and chronic kidney disease, demonstrating that the patients with T2DM and moderate/severe periodontal disease had a statistically significant higher risk of microalbuminuria and end-stage renal disease, with an incidence of microalbuminuria being 2 times higher, and an incidence of end-stage renal disease being 2 to 5 times higher compared to the patients with T2DM with mild/absent periodontal disease [4,38]. Although in our study we could not prove a statistically significant association between the presence of chronic kidney disease and complicated dental lesions, we did observe that these patients presented a higher frequency of chronic kidney disease compared to the patients with a superficial lesion. However, the analysis of the eGFR showed that patients with complicated lesions had lower eGFR values that were statistically significant, with decreased eGFR being a predictor for the presence of complicated dental lesions in the patients with T2DM enrolled in our study.

In interpreting our study results, we cannot overlook the finding of Nitta et al. Their cross-sectional study that included 620 patients with T2DM demonstrated statistically significant associations between periodontal disease and microvascular complications, as well as associations between periodontal disease and glycemic control [39]. The authors reported that while the number of diabetes microvascular complications was correlated with the severity of periodontal disease in T2DM, there was no evidence of a statistically significant association between microvascular complications and the frequency of periodontal disease [39]. Another important finding of this study was the association of poor glycemic control evidenced by the HbA1c value ≥ 8.0% with both severity and higher frequency of periodontitis [39]. Our study confirms these findings and proposes a score that can be used in clinical practice as it is predictive for the presence of complicated dental and periodontal lesions in patients with T2DM.

Our finding regarding the associations of poor glycemic control with dental lesions is also supported by a paper published in 2022 that demonstrated that patients with T2DM with HbA1c ≥ 8.0% had a significantly higher frequency of decayed teeth [15].

The limitations of the study are represented by the fact that our study was carried out in a limited geographical area; therefore, the results cannot be extrapolated to the general population. Furthermore, the small sample size that was not suitable for multivariate regression analysis represents another limitation of our study. However, the data obtained can be applied to populations with similar characteristics to the subjects in the study. Thus, dental and periodontal lesions should be considered an important modifiable risk factor in subjects with T2DM, with education and dental care being extremely important. Another limitation of this study was represented by the lack of a group of subjects with T2DM without dental and periodontal lesions, but this study was performed in hospitalized patients in whom we did not identify subjects without any lesions. Despite all these limitations, the obtained results indicate that the frequent evaluation of dental and periodontal lesions, but also of the chronic complications of T2DM, is imperative to reduce the incidence of these cases.

## 5. Conclusions

Various oral lesions are present at a high frequency among subjects with T2DM, a fact that will negatively influence their quality of life. Our study demonstrated that the development of complicated dental and periodontal lesions in patients with T2DM can be predicted both by the existence of chronic microvascular complication as well as by the abnormal values of parameters associated with glycemic control (fasting blood plasma and HbA1c) and chronic inflammation, particularly fibrinogen. We propose an easily calculable score with four items that can predict the risk of complicated dental and periodontal lesions in patients with T2DM, which in clinical practice can be beneficial to diabetologists and dentists alike. However, further studies are needed for external validation of this score.

## Figures and Tables

**Table 1:** Demographic, anthropometric and paraclinical characteristics of the studied patients.

Characteristic		Total	Control Group (Superficial Lesions)	Study Group (Complicated Lesions)	*p* Value
Gender (no; %)	Females	54 (50%)	32 (59.3%)	33 (61.1%)	0.844
	Males	54 (50%)	22 (40.7%)	21 (38.9%)	
Age (years)		62.54 ± 6.79	61.69 ± 5.41	63.39 ± 7.89	0.194
BMI (kg/m^2^)		30.39 [6.79]	31.37 [6.68]	30.05 [6.37]	0.699
Nutritional status (no; %)	Normal weight	12 (11.1%)	9 (16.7%)	3 (5.6%)	0.231
	Overweight	36 (33.3%)	15 (27.8%)	21 (38.9%)	
	Class 1 obesity	36 (33.3%)	18 (33.3%)	18 (33.3%)	
	Class 2 obesity	15 (13.9%)	6 (11.1%)	9 (16.7%)	
	Class 3 obesity	9 (8.3%)	6 (11.1%)	3 (5.6%)	
Fasting blood glucose (mg/dL)		152 [66.25]	144 [37]	200 [88]	<0.001
HbA1c (%)		7.65 [1.05]	7.45 [1.1]	8 [1]	<0.001
Creatinine (mg/dL)		0.8 [0.23]	0.76 [0.19]	0.85 [0.24]	0.031
eGFR (mL/min/1.73 m^2^)		93.7 [33.45]	96.7 [19.9]	89.5 [38.3]	0.016
Albuminuria (no; %)		16 (14.8%)	5 (9.3%)	11 (20.4%)	0.104
ESR (mm/h)		32 [23.75]	28.5 [12]	46.5 [30]	0.001
C-reactive protein (mg/dL)		0.7 [0.31]	0.7 [0.22]	0.69 [0.84]	0.761
Fibrinogen (mg/dL)		255.5 [120.5]	196 [41]	308 [100]	<0.001
T2DM treatment (no; %)	Insulin	35 (32.4%)	8 (14.8%)	27 (50%)	<0.001
	Metformin	90 (83.3%)	52 (96.3%)	38 (70.4%)	<0.001
	Sulfonylurea	33 (30.6%)	17 (31.5%)	16 (29.6%)	0.835
	DPP-4 i	5 (4.6%)	2 (3.7%)	3 (5.6%)	0.647
	GLP-1 RA	19 (17.6%)	5 (9.3%)	14 (25.9%)	0.023
	SGLT2 i	14 (13.0%)	9 (16.7%)	5 (9.3%)	0.252
T2DM duration from diagnosis (years)		7.5 [10]	7 [7]	9 [9]	0.056

BMI: body mass index; DPP-4 i: dipeptidyl peptidase 4 inhibitors; eGFR: estimated glomerular filtration rate; ESR: erythrocyte sedimentation rate; GLP-1 RA: glucagon like peptide 1 receptor agonists; HbA1c: glycated hemoglobin; SGLT2 i: sodium–glucose cotransporter-2 inhibitors; T2DM: type 2 diabetes mellitus. Continuous variables with abnormal distribution are presented as median [IQR].

**Table 2:** The frequency of microvascular complications in the study groups.

Microvascular Complications	Total	Control Group (Superficial Lesions)	Study Group (Complicated Lesions)	*p* Value
Diabetic retinopathy (no; %)	25 (23.1%)	8 (14.8%)	17 (31.5%)	0.040
Sensory-motor peripheral diabetic neuropathy (no; %)	82 (75.9%)	33 (61.1%)	49 (90.7%)	<0.001
Chronic kidney disease (no; %)	17 (15.7%)	5 (9.3%)	12 (22.2%)	0.064

**Table 3:** The AUROCs of tested variables as predictors for complicated dental and periodontal lesions.

Variable	AUROC	Standard Error	Significance *p* Value	95% CI
Fibrinogen (mg/dL)	0.836	0.038	<0.001	0.762–0.910
Fasting plasma glucose (mg/dL)	0.807	0.042	<0.001	0.724–0.889
HbA1c (%)	0.708	0.049	<0.001	0.612–0.804
ESR (mm/h)	0.685	0.056	0.001	0.576–0.794
Creatinine (mg/dL)	0.620	0.054	0.031	0.515–0.726
eGFR (mL/min/1.73 m^2^) *	0.366	0.055	0.016	0.258–0.473

* Inverse association; eGFR: estimated glomerular filtration rate; ESR: erythrocyte sedimentation rate; HbA1c: glycated hemoglobin.

**Table 4:** Scoring table for the score associated with complicated dental and periodontal lesions in patients with T2DM.

Items	0 Points	1 Point
Fibrinogen (mg/dL)	<240 mg/dL	≥240 mg/dL
Fasting plasma glucose (mg/dL)	<149 mg/dL	≥149 mg/dL
Diabetic retinopathy	Absent	Present
Diabetic peripheral neuropathy	Absent	Present

## Data Availability

All the data are contained in the manuscript.
